# Supplementary material for: Development of Highly Efficient Resistance to Beet Curly Top Iran Virus (Becurtovirus) in Sugar Beet (B. vulgaris) via CRISPR/Cas9 System
Source: Int J Mol Sci. 2023 Mar 30;24(7):6515. doi: 10.3390/ijms24076515 (PMC10095410; doi:10.3390/ijms24076515)
Supplement: Supplementary file 1 [file ijms-24-06515-s001.zip › Supp.File S1_gRNA seqeunce.pdf]

Supplemantary file S1: The sequence of gRNAs and the restriction enzyme cutting site at their target site with their GC content and folding harmony with tracRNA.

NAME	TARGET SITE (PAM)	GC%	RE SITES	gRNA - tractrRNA Folding	Targeted gene
**1. gRNA_C1**	TTGCTGTTGCCAATACTGTTTGG	40	HpyCH4III	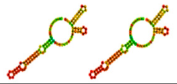	**C1-REPLICATION PROTEİN A (REP-A)**
**2. gRNA_C1./C2**	GCCTAGAGATCTTGCCCACTGGG	55	BtsIMutI	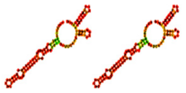	**C1/C2- INTRONIC REGION BETWEEN REPLICATION PROTEİN A and B**
**3. gRNA_C2**	GCAATACAAGGTCTTCCGCCTGG	55	AciI	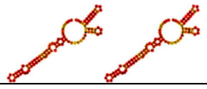	**C2- REPLICATION PROTEİN B (REP-B)**
**4. gRNA_V1**	CCTTCCACTTATCGGATTAGGAG	45	BslI	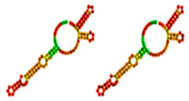	**V1-CAPSID PROTEIN (CP)**
**5. gRNA_V1/V2**	GGAAGAGGAAGACGACCGGTGGG	60	AgeI	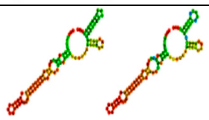	**V1/V2- OVERLAPPING REGION BETWEEN CAPSID AND MOVEMENT PROTEIN**
**6. gRNA_V2**	GCGTAAGTGTAGTTTGTACGAGG	45	RsaI	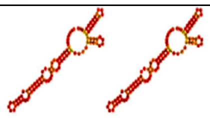	**V2-MOVEMENT PROTEIN (MP)**
**7.gRNA_V2/V3**	CCTTTCAGAGTGAGCGAATTTCC	45	Hpy188I	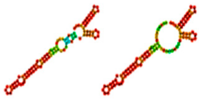	**V3/V2 – OVERLAPING REGION BETWEEN ssDNA REGULATOR AND MOVEMENT PROTEIN**
**8. gRNA_V3**	TGATGGTGTGTATTCCCGACTGG	50	Hpy188III	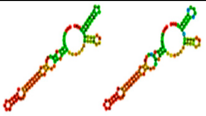	**V3-ssDNA REGULATOR PROTEIN**
