# Supplementary material for: Development of Highly Efficient Resistance to Beet Curly Top Iran Virus (Becurtovirus) in Sugar Beet (B. vulgaris) via CRISPR/Cas9 System
Source: Int J Mol Sci. 2023 Mar 30;24(7):6515. doi: 10.3390/ijms24076515 (PMC10095410; doi:10.3390/ijms24076515)
Supplement: Supplementary file 1 [file ijms-24-06515-s001.zip › Supplementary file S2_multiplex.pdf]

Supplemantary File S2: Experimental procedure for multiplex CRISPR targeting all functional genes of BCTIV genes named as; C1 (RepA), C2(RepB) and overlapping region between V1/V2(CP-MP) and V2/V3 (MP/ssDNA).

Supp. File_Table S1: gRNA sequences for multiplex CRISPR

gRNA	Sequence
DT1-BsF: 3. gRNA_C1	5'- AATAATGGTCTCTATTGTTGCTGTTGCCAATACTGTTGTT-3'
DT1-F0: 3. gRNA_C1	5'- TGTTGCTGTTGCCAATACTGTTGTTTTAGAGCTAGAAATAGC -3'
DT2-BsF2: 15.gRNA_V1/V2	5' AATAATGGTCTCTAGATTGGGAAGAGGAAGACGACCGGTGTT 3'
DT2-F0: 15.gRNA_V1/V2	5'- TGGGAAGAGGAAGACGACCGGTGTTTTAGAGCTAGAAATAGC -3'
DT3-BsF3: 6. gRNA_C2	5'- AATAATGGTCTCTGTGATTGGCAATACAAGGTCTTCCGCCGTT -3'
DT3-F0: 6. gRNA_C2	5'- TGGCAATACAAGGTCTTCCGCCGTTTTAGAGCTAGAAATAGC -3'
DT4-R0: 19. gRNA_V3/V2	5'-AACTTCAGAGTGAGCGAATTTCCCAATCACTACTTCGACTCTAGCTGTAT -3'
DT4-BsR: 19. gRNA_V3/V2	5'- ATTATTGGTCTCTAAACTTCAGAGTGAGCGAATTTCC -3'

Supp. File_Table S2. PCR conditions for clonning of gRNA_C1 by using pCBC-DT1T2 vector

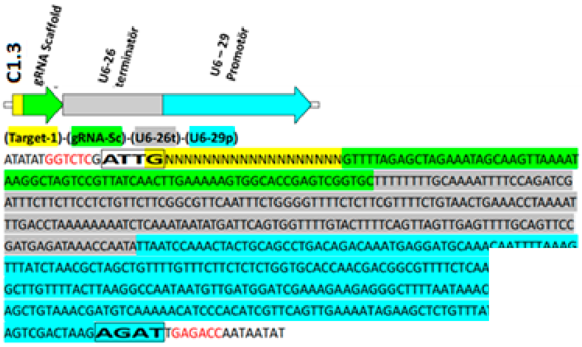

Components	(µl)
10× Q5 polimeraz Buffer	5
MgSO4 (25mM)	3
dNTPs (2mM)	4
Q5 polimeraz	1
pCBC-DT1T2 (diluted to 200 times) plazmid (adgene 50590)	1
DT1-BsF (20 µM) gRNA C1.3	1
DT1-F0 (1 µM) gRNA C1.3	1
DT0-BsR (20 µM) primer	1
ddH2O	33
Total volume	50 ul

PCR conditions

1. One cycle: 98 °C, 30 sec.

2. 34 cycles: 98 °C, 10 sec; 65 °C, 30 sec; 72 °C, 30 sec.

3. One cycle: 72 °C, 7 min

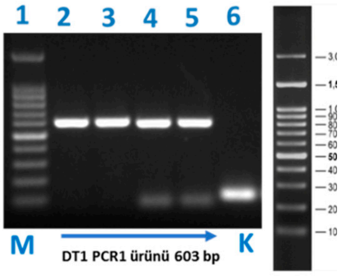

Supp. File_Table S3. PCR conditions for clonning of gRNA_ V1/V2 by using pCBC-DT2T3

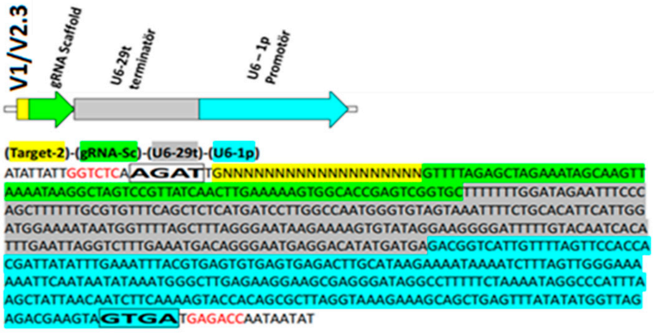

Bileşenler	µl	Döngü koşulları
10× Q5 polimeraz enzim Buffer	5	
MgSO4 (25mM)	3	
dNTPs (2mM)	4	**1.** One cycle: 98 °C, 30 sec.
Q5 polimeraz enzim	1	
pCBC-DT1T2 (diluted to 200 times) plazmid (adgene 50590)	1	**2.** 34 cycles: 98 °C, 10 sec; 65 °C, 30 sec; 72 °C, 30 sec.
DT2-BsF (20 µM) gRNA V1/V2.3	1	
DT2-F0 (1 µM) gRNA V1/V2.3	1	**3.** One cycle: 72 °C, 7 min
DT0-BsR3 (20 µM)	1	
ddH2O	33	
Total hacim	50 ul	

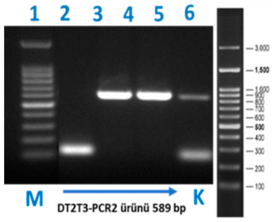

Supp. File_Table S4. PCR conditions for clonning of gRNA_C2 and gRNA_V3/V2 by using pCBC-DT3T4 vector

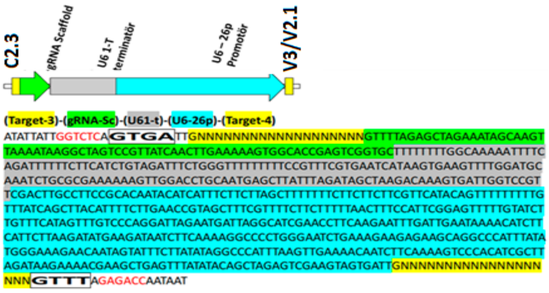

Components	ul	Cycle
10× Q5 polimerase enzym Buffer	5	
MgSO4 (25mM)	3	
dNTPs (2mM)	4	**1.** One cycle: 98 °C, 30 sec.
Q5 polimerase enzim	1	
pCBC-DT1T2 (diluted to 200 times) plazmid (adgene 50590)	1	**2.** 34 cycles: 98 °C, 10 sec; 65 °C, 30 sec; 72 °C, 30 sec.
DT3-F0 (1 µM) gRNA C2.3	1	
DT4-R0: (1 µM) gRNAV3/V2.1	1	**3.** One cycle: 72 °C, 7 min
DT4-BsR (20 µM) gRNAV3/V2.1	1	
ddH2O	32	
Total	50 ul	

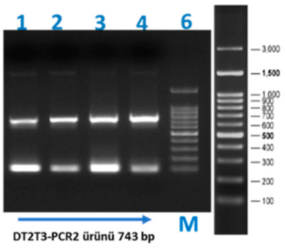

DT3T4-PCR3 ürünlerinin jel görüntüsü Lane 6: 100 bp markör (Biomol). Lane 1-4 [illegible]CR ürünü.

Supp.File_ Table S5. Golden gate reaction with three PCR fragments to clone them into pHSE401 vector

Components	Quantity (ul)	Cycle
Purified DT1 PCR1 fragments (~17 ng/µl)	4	
Purified DT2T3-PCR2 fragments (~12 ng/µl)	5	
Purified DT3T4-PCR3 fragments (~11 ng/µl)	6	
pHSE401 (~160 ng/µl)	2	**1. 30 cycles** 5 min at 37°C
10× T4 DNA Ligase Buffer (NEB)	1.5	2. **30 cycles** 5 min at 16 °C
10× BSA	1.5	**2.** 5 min at 55°C
BsaI (NEB)	1	**3.** 5 min at 80°C
T4 DNA Ligase (HC, NEB)	1	
ddH2O	3	
Total volume	25	

Supp. File_ Table S6. Verification of cloning into pHSE401 and transfer into *E.coli* and *Agrobacterium* with colony PCR

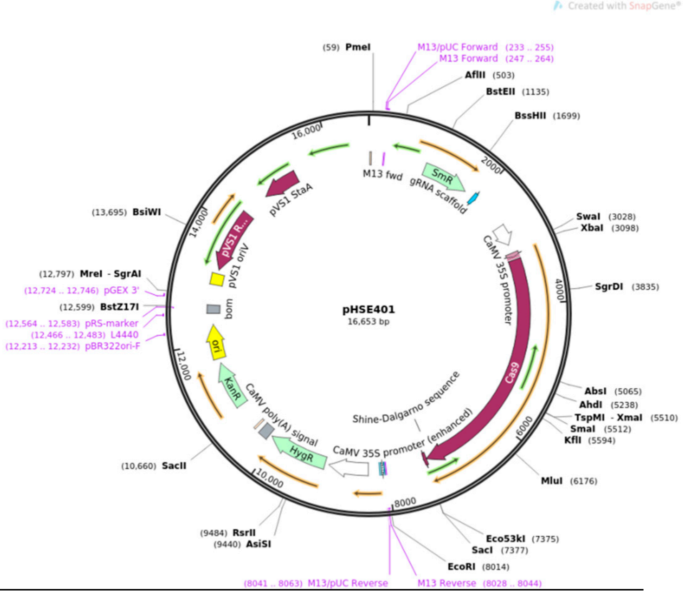

PCR Components		Colony PCR Protocol		
**Components**	**Quantity**	Temperature	Time	Cycle
NEB 2x Taq DNA	12.5 ul	95 °C	3 min	1
Master mix		95 °C	30 sec	34
U6-29p-F	1 ul	55	30 sec	34
U6-1t-R	1 ul	68 °C	1 min	34
Nuclease free water	10.5 ul	68 °C	5 min	1
DNA	-	4 °C		Store
(taken from colony				
by the tip of pipette)				
**Total**	**25 ul**			

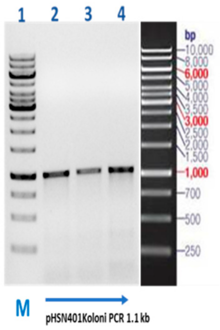

U6-29p-F: TTAATCCAAACTACTGCAGCCTGAC and U6-1t-R: AACGGACCAATCACTTTGTCTTAGC
