# Supplementary material for: Development of Highly Efficient Resistance to Beet Curly Top Iran Virus (Becurtovirus) in Sugar Beet (B. vulgaris) via CRISPR/Cas9 System
Source: Int J Mol Sci. 2023 Mar 30;24(7):6515. doi: 10.3390/ijms24076515 (PMC10095410; doi:10.3390/ijms24076515)
Supplement: Supplementary file 1 [file ijms-24-06515-s001.zip › Supplementary file S3_ mutation analysis.pdf]

Supp.file S3: gRNA/Cas9-mediated mutations in BCTIV genome detected by PCR-RE assay and sanger sequencing. Red arrows indicate non-cut fragments due to the mutation in the RE cleavage site created by gRNA/Cas9 system in the virus genome. WT: fragments of virus obtained from only BCTIV agroinoculated sugar beets; MUT: viral bands formed by RE cutting of PCR fragments obtained from co-agroinoculated sugar beet with gRNA/Cas9 and BCTIV. Sequence of mutated viral genome was analysed with Tracking of Indels by DEcomposition (TIDE) program.

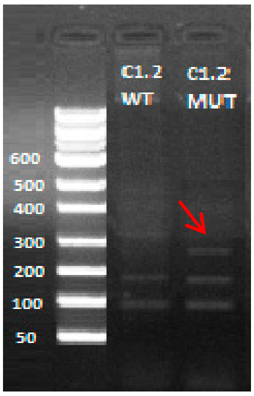

TIDE analysis of viral sequence for the gRNA/Cas9 treated REPA region (C1)

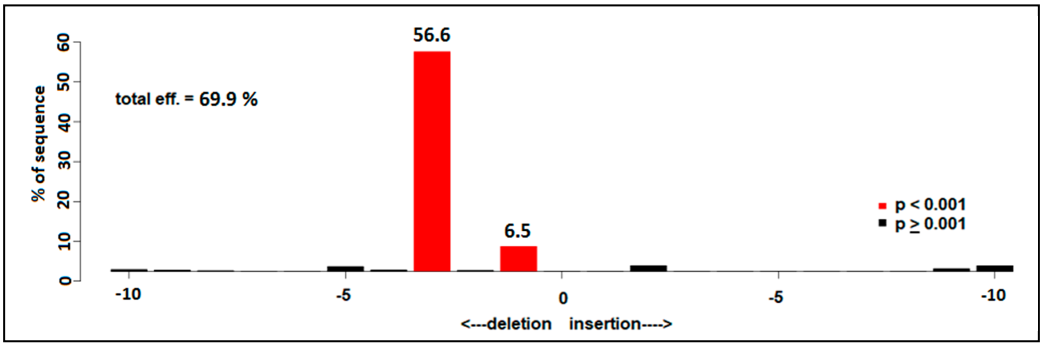

Mutation in gRNA/Cas9 targeted viral REPA (C1) that was not digested with HpyCH4III

HpyCH4III

TCCTTGGTGTTGGATGAATCAAGAATTCGTCTCCATTCCATGTTGCTGTTGCCAATACTGTTTGGAGAGCGCCCTCCCATCG WT

TCCTTGGTGTTGGATGAATCAAGAATTCGTCTCCATTCCATGTTGC<<<<<<<<<<<<<<<<<TTGGAGAGCGCCCTCCCATCG -17

TCCTTGGTGTTGGATGAATCAAGAATTCGTCTCCATTCCATGTTGCTGTTGCCAA<<<<GTTTGGAGAGCGCCCTCCCATCG -8

TCCTTGGTGTTGGATGAATCAAGAATTCGTCTCCATTCCATGTTGCTGTTGCCAATACgcTGTTTGGAGAGCGCCCTCCCATCG +2

TIDE analysis of viral sequence for the gRNA/Cas9 treated overlapping region between REPA/REPB

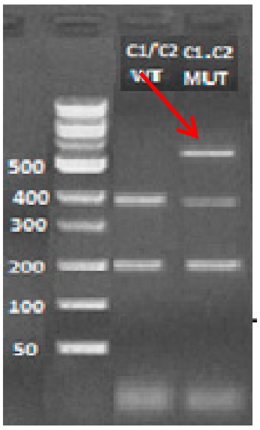

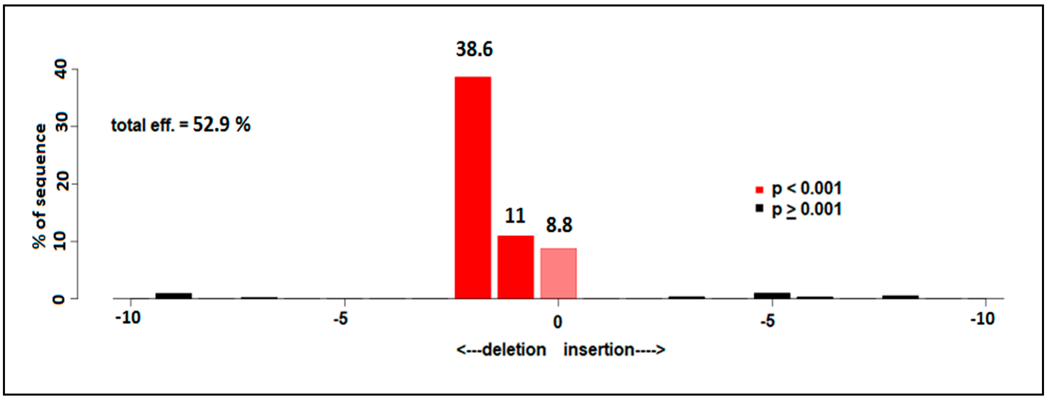

Mutation in gRNA/Cas9 targeted viral REPA/REPB (C1/C2) that was not digested with BtsIMutI

BtsIMutI

GAGACCAATCTAATACCATGCCAGACATATAGTGGTGTCGGCCTAGAGATCTTGCCCACTGGGTCTTTCCTGTTCTGGAT WT

GAGACCAATCTAATACCATGCCAGACATATAGTGGTGTCGGCC<<<<<<<<<<<<<<<<<<<<<<< CTTTCCTGTTCTGGAT -23

GAGACCAATCTAATACCATGCCAGACATATAGTGGTGTCGGC<<<<<<<<<<<<<<< CACTGGGTCTTTCCTGTTCTGGAT -15

GAGACCAATCTAATACCATGCCAGACATATAGTGGTGTCGGCCTAGAGATCTT<<<<< CTGGGTCTTTCCTGTTCTGGAT -4

GAGACCAATCTAATACCATGCCAGACATATAGTGGTGTCGGCCTAGAGATCTTGtgaCCCACTGGGTCTTTCCTGTTCTGGAT +3

GAGACCAATCTAATACCATGCCAGACATATAGTGGTGTCGGCCTAGAGATCTTGCCGTCTGGGTCTTTCCTGTTCTGGAT G>C/T>A

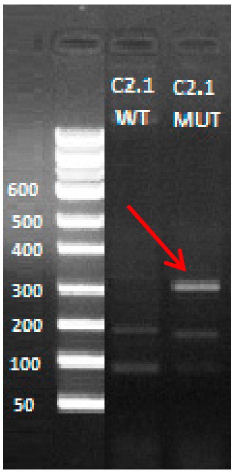

TIDE analysis of viral sequence for the gRNA/Cas9 treated REPB (C2)

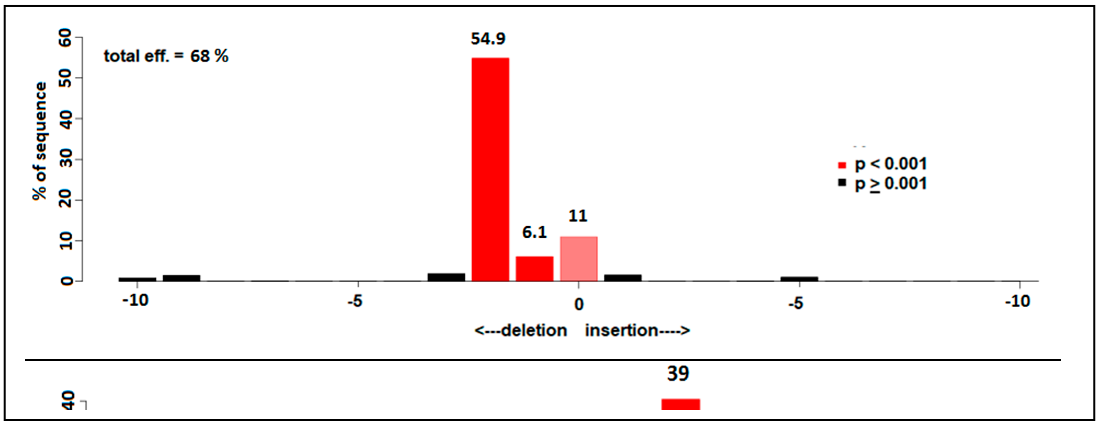

Mutation in gRNA/Cas9 targeted viral REPB (C2) that was not digested with AciI

AciI
CATAGATTCACTCATACAAGGTATCCAGTCCATATCTGGATTAGTAATAGCAATACAAGGTCTTCCGCCTGGAATGACTAAGT
CATAGATTCACTCATACAAGGTATCCAGTCCATATCTGGAT<<<<<<<<<<<<<<<<<<<<<<<<<<< GCCTGGAATGACTAAGT -27
CATAGATTCACTCATACAAGGTATCCAGTCCATATCTGGATTAGTAATAGCAATACAAG<<<<<<<<< CTGGAATGACTAAGT -9
CATAGATTCACTCATACAAGGTATCCAGTCCATATCTGGATTAGTAATAGCAAT<<<<<<<<<<<<<<<<<<<<AATGACTAAGT -20
CATAGATTCACTCATACAAGGTATCCAGTCCATATCTGGATTAGTAATAGCAATACAAGGTCAACCGCCTGGAATGACTAAGT A<T

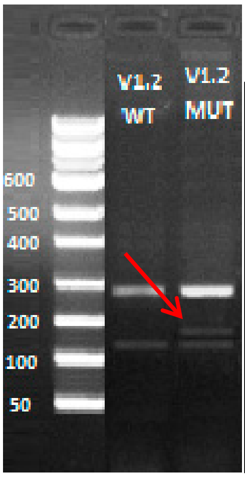

TIDE analysis of viral sequence for the gRNA/Cas9 treated Capsid gene (V1)

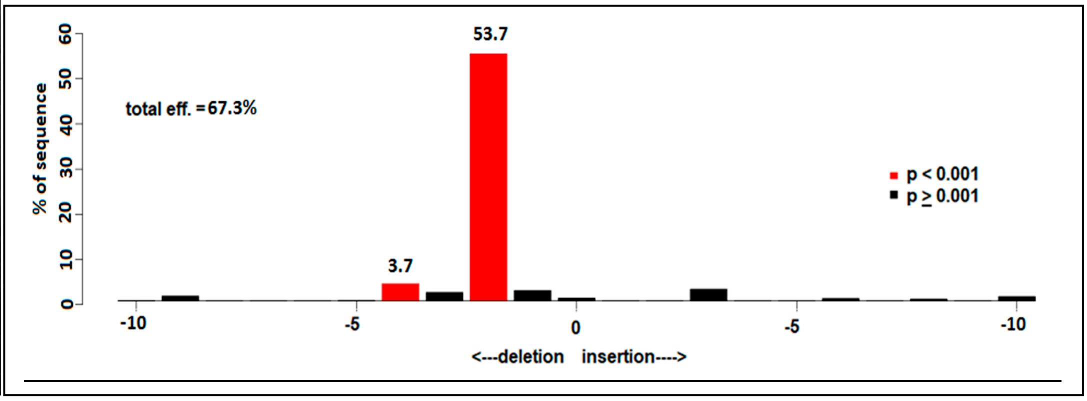

Mutation in gRNA/Cas9 targeted viral CP (V1) that was not digested with BslI

BslI
GACTGGTGGTCAGGCTATGCCTTCCACTTATCGGATTAGGAGGGATGCGAATGAACGATTTATCGTGAAGAAGAAGTG WT
GACTGGTGGTCAGGCTATGCCTTCC<<<<<<<<<<<TTAGGAGGGATGCGAATGAACGATTTATCGTGAAGAAGAAGTG -11
GACTGGTGGTCAGGCTATGCCTT<<<<<<ATCGGATTAGGAGGGATGCGAATGAACGATTTATCGTGAAGAAGAAGTG -6
GACTGGTGGTCAGGCTATGCCTTCC<<TTATCGGATTAGGAGGGATGCGAATGAACGATTTATCGTGAAGAAGAAGTG -2
GACTGGTGGTCAGGCTATGCCTTCCtgttACTTATCGGATTAGGAGGGATGCGAATGAACGATTTATCGTGAAGAAGAAGTG +4

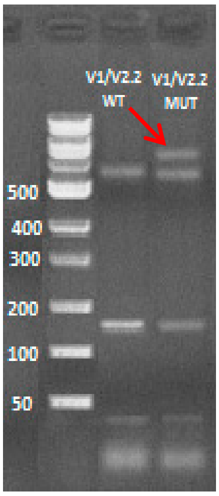

TIDE analysis of viral sequence for the gRNA/Cas9 treated overlapping region between capsid and movement gene (V1/V2)

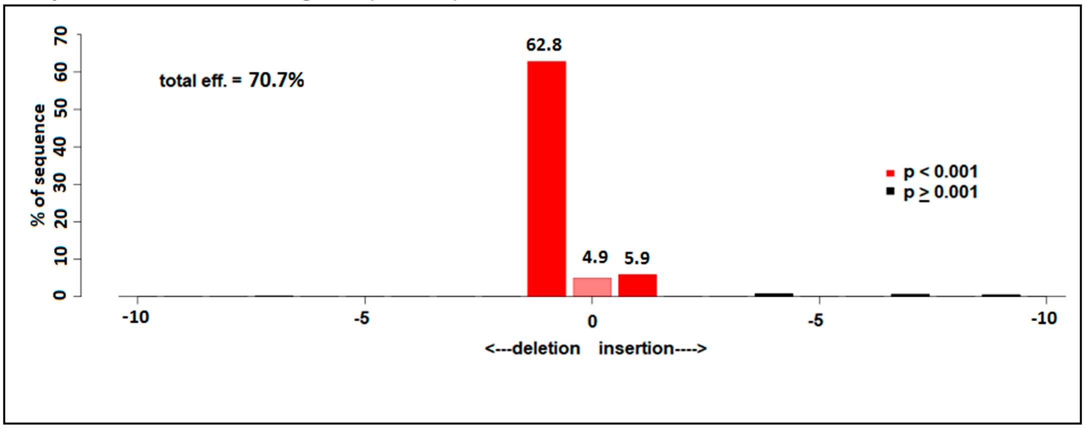

Sequences of gRNA/Cas9 targeted overlapping CP/MP region (V1/V2.2) that was not cleaved by AgeI

AgeI
AAAAGAGAAAGTATACTCCTCCGGCCAGTTGGACGAGGAAGAGGAAGACGACCGGTGGGAGGACAGTTTCGAAGAA WT
AAAAGAGAAAGTATACTCCTCCGGCCAGTTGGACGA<<<<<<<<<<<<<<<<<<<<GGTGGGAGGACAGTTTCGAAGAA -20
AAAAGAGAAAGTATACTCCTCCGGCCAGTTGGACGAGGAAGAGGA<<<<<<<<< GGTGGGAGGACAGTTTCGAAGAA -9
AAAAGAGAAAGTATACTCCTCCGGCCAGTTGGACGAGGAAGAGGAAGA<<<<<<<<<< GGAGGACAGTTTCGAAGAA -10
AAAAGAGAAAGTATACTCCTCCGGCCAGTTGGACGAGGAAGAGGAAGggg<<<<GGTGGGAGGACAGTTTCGAAGAA -4/+3

TIDE analysis of viral sequence for the gRNA/Cas9 treated movement gene (V2)

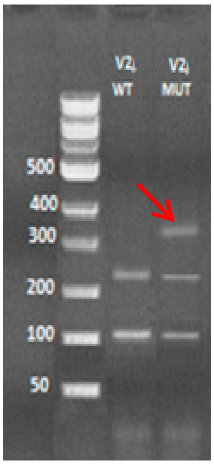

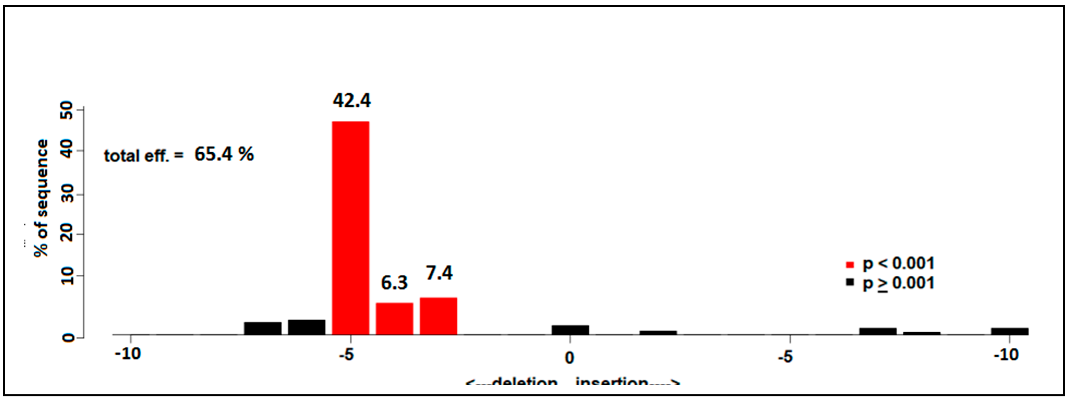

Sequences of gRNA/Cas9 targeted overlapping CP/MP region (V2) that was not cleaved by RsaI

RsaI
GGAAGTGAAGAAATTGCTGAAGCTTAAGGTTAGTTTTAAGCGTAAGTGTAGTTTGTACGAGGACATATACAAGAAGTAT
GGAAGTGAAGAAATTGCTGAAGCTTAAGGTTAGTTTTAAGCGTAA<<<<<<<<<<<<ACGAGGACATATACAAGAAGTAT -12
GGAAGTGAAGAAATTGCTGAAGCTTAAGGTTAGTTTTAAGCGTAAGTGTAGT<<<<<<< AGGACATATACAAGAAGTAT -7
GGAAGTGAAGAAATTGCTGAAGCTTAAGGTTAGTTTTAAGCGTAAGTGTAGTTTGgtTACGAGGACATATACAAGAAGTAT +2

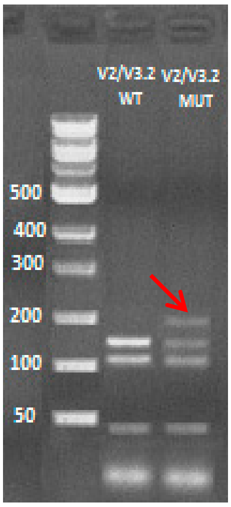

TIDE analysis of viral sequence for the gRNA/Cas9 treated overlapping region between movement and ssDNA regulator gene (V1/V2)

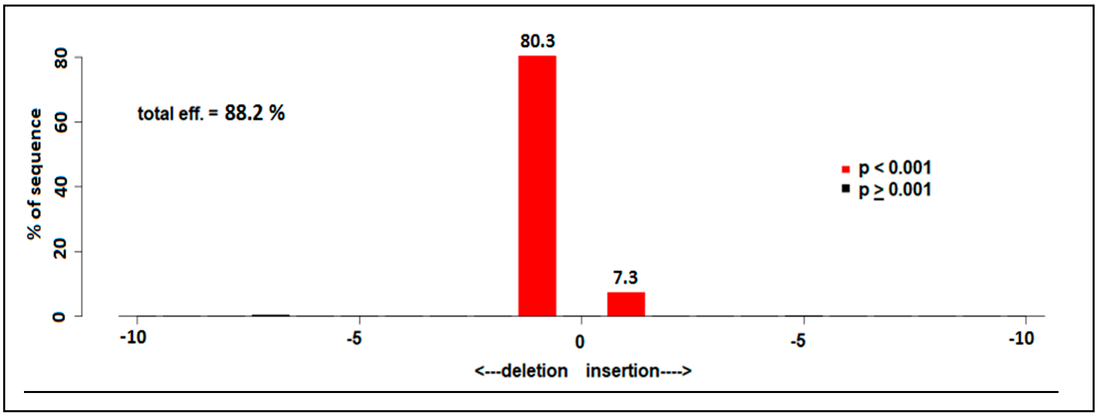

Sequences of gRNA/Cas9 targeted overlapping MP/ssDNA region (V2/V3) that was not cleaved by Hpy188I

Hpy188I

ATTTAGTACGATTCTTCAGTCGGGAATTAATTTCTATGGTACCTTTCAGAGTGAGCGAATTTCCGCGAAGCTATCCAGC	
ATTTAGTACGATTCTTCAGTCGGGAATTAATTTCTATGGTAC<<<<<<<<<<<<<<<<<< ATTTCCGCGAAGCTATCCAGC	-18
ATTTAGTACGATTCTTCAGTCGGGAATTAATTTCTATGGTACCTTTCAGAG<<<<<<<< ATTTCCGCGAAGCTATCCAGC	-8
ATTTAGTACGATTCTTCAGTCGGGAATTAATTTCTATGGTACCTTTCAGAGTGA<<<gggc TTCCGCGAAGCTATCCAGC	-3/+4
ATTTAGTACGATTCTTCAGTCGGGAATTAATTTCTATGGTACCTTTCAGAGTGAGCGTATTTCCGCGAAGCTATCCAGC	T<A
ATTTAGTACGATTCTTCAGTCGGGAATTAATTTCTATGGTACCTTTCAGAGTGAGCGTaatATTTCCGCGAAGCTATCCAGC	+3

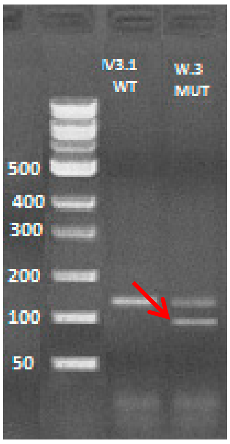

TIDE analysis of viral sequence for the gRNA/Cas9 treated ssDNA regulator gene (V3)

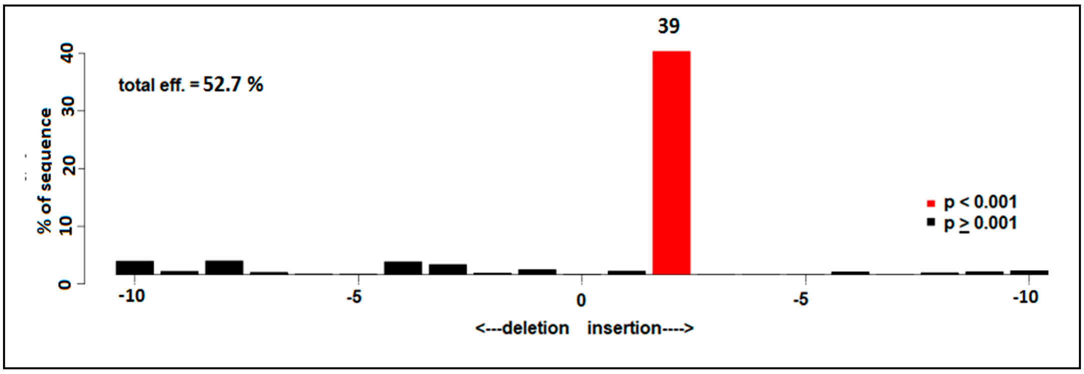

Sequences of gRNA/Cas9 targeted ssDNA regulator region (V3) that was not cleaved by Hpy188III

Hpy188III

CGTATATTATACGTATAAATGATGGTGTGTATTCCCGACTGGTTATTTCTACTTTTCGTATTTAGTACGATTCTTCAG	WT
CGTATATTATACGTATAAATGATGGTG<<<<<<<<<<<ACTGGTTATTTCTACTTTTCGTATTTAGTACGATTCTTCAG	-11
CGTATATTATACGTATAAATGATGGTGTGTATT<<<<<<< GGTTATTTCTACTTTTCGTATTTAGTACGATTCTTCAG	-7
CGTATATTATACGTATAAATGATGGTGTGTATTC<<< ACTGGTTATTTCTACTTTTCGTATTTAGTACGATTCTTCAG	-3
CGTATATTATACGTATAAATGATGGTGTGTATTC<<gggGACTGGTTATTTCTACTTTTCGTATTTAGTACGATTCTTCAG	-2/C<G
